# Interaction of Pattern Recognition Receptors with Mycobacterium Tuberculosis

**DOI:** 10.1007/s10875-014-0103-7

**Published:** 2014-10-14

**Authors:** Esmaeil Mortaz, Ian M. Adcock, Payam Tabarsi, Mohammad Reza Masjedi, Davood Mansouri, Ali Akbar Velayati, Jean-Laurent Casanova, Peter J. Barnes

**Affiliations:** 1 Division of Pharmacology and Pathophysiology, Utrecht Institute for Pharmaceutical Sciences, Faculty of Sciences, Utrecht University, Utrecht, The Netherlands; 2 Cell and Molecular Biology Group, Airways Disease Section, National Heart and Lung Institute, Faculty of Medicine, Imperial College London, Dovehouse Street, London, SW3 6LY UK; 3 Clinical Tuberculosis and Epidemiology Research Center, National Research and Institute of Tuberculosis and Lung Diseases (NRITLD), Shahid Beheshti University of Medical Sciences, Tehran, Iran; 4 Chronic Respiratory Diseases Research Center and National Research Institute of Tuberculosis and Lung Diseases (NRITLD), Shahid Beheshti University of Medical Sciences, Tehran, Iran; 5 Howard Hughes Medical Institute and St. Giles Laboratory of Human Genetics of Infectious Diseases, The Rockefeller University, New York, 10065 NY USA; 6 Paris Descartes Sorbonne Paris Cité University, Imagine Institute, Paris, France; 7 Laboratory of Human Genetics of Infectious Diseases, INSERM UMR 1163, Imagine Institute, Necker Hospital for Sick Children, Paris, France; 8 Pediatric Hematology and Immunology Unit, Necker Hospital for Sick Children, AP-HP, Paris, France

**Keywords:** Tuberculosis, TLRs, inflammasome

## Abstract

*Tuberculosis* (TB) is considered a major worldwide health problem with 10 million new cases diagnosed each year. Our understanding of TB immunology has become greater and more refined since the identification of Mycobacterium tuberculosis (MTB) as an etiologic agent and the recognition of new signaling pathways modulating infection. Understanding the mechanisms through which the cells of the immune system recognize MTB can be an important step in designing novel therapeutic approaches, as well as improving the limited success of current vaccination strategies. A great challenge in chronic disease is to understand the complexities, mechanisms, and consequences of host interactions with pathogens. Innate immune responses along with the involvement of distinct inflammatory mediators and cells play an important role in the host defense against the MTB. Several classes of pattern recognition receptors (PRRs) are involved in the recognition of MTB including Toll-Like Receptors (TLRs), C-type lectin receptors (CLRs) and Nod-like receptors (NLRs) linked to inflammasome activation. Among the TLR family, TLR1, TLR2, TLR4, and TLR9 and their down-stream signaling proteins play critical roles in the initiation of the immune response in the pathogenesis of TB. The inflammasome pathway is associated with the coordinated release of cytokines such as IL-1β and IL-18 which also play a role in the pathogenesis of TB. Understanding the cross-talk between these signaling pathways will impact on the design of novel therapeutic strategies and in the development of vaccines and immunotherapy regimes. Abnormalities in PRR signaling pathways regulated by TB will affect disease pathogenesis and need to be elucidated. In this review we provide an update on PRR signaling during *M. tuberculosis* infection and indicate how greater knowledge of these pathways may lead to new therapeutic opportunities.

## Introduction

Tuberculosis (TB) is one of the most common infections worldwide, and in 2012, an estimated 8.6 million people developed TB and 1.3 million died from the disease (including 320,000 deaths among HIV-positive people) [1, 2]*. Mycobacterium tuberculosis* (MTB) is an intracellular pathogen capable of infecting and surviving within the host’s mononuclear cells particularly macrophages. This involves sequestration of MTB within organized granulomas. Elimination of the microorganism is through a combination of various killing mechanisms including apoptosis of host macrophages [3]. These responses are orchestrated by T helper 1-type (Th1) pro-inflammatory cytokines, which are synthesized by phagocytes upon recognition of pathogen-associated molecular patterns (PAMPs) on MTB by pattern recognition receptors (PRRs). MTB is usually transmitted via aerosols and establishes a stable infectious state in the respiratory system. There, MTB is engulfed by macrophages and dendritic cells (DCs), which serve as host cells for MTB survival and propagation [4]. Binding of MTB ligands to TLR-2, -4 and -9 initiates release of inflammatory mediators, expression of adhesion molecules and further recruitment of macrophages, DCs and PMN to the MTB infected area [5].

**Fig. 1:**
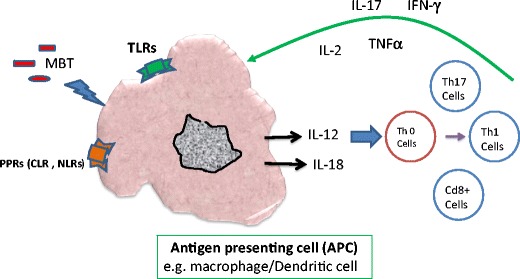
Schematic diagram indicating the role of specific cell types and mediators on the induction of IFNγ by MTB and the subsequent killing of macrophage-resident bacteria. *Abbreviations*: *CLR* C-type lectin receptors, *MTB* Mycobacterium tuberculosis, *TLRs* Toll like receptors, *NLRs* NOD-like receptors

**Fig. 2:**
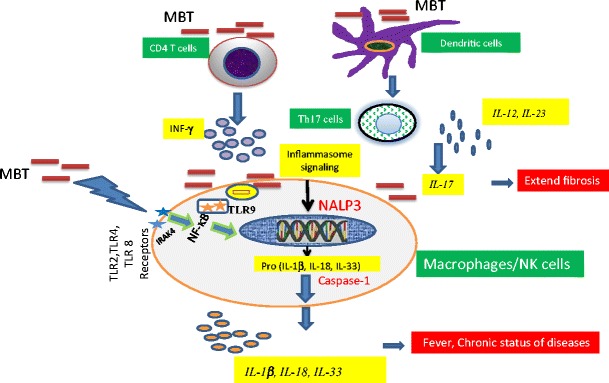
The putative role of TLR/Inflammasome signaling on the regulation of MTB in the cells. *Abbreviation*: *TLR* Toll like receptor, *MTB* Mycobacterium tuberculosis

Although the host’s innate immune response to *MTB* infection is critical for the initial defense against bacteria, the adaptive immune response is ultimately required for containment of the infection in the chronic stage of disease. Adaptive immunity to MTB infection is characterized by the appearance of antigen specific CD4+ T-cells that secrete IFN-γ, which, in turn, activates macrophages and other antigen presenting cells (APC) to kill intracellular bacteria [6]. CD8+ T-cells are also important cells for controlling MTB during the chronic phase of infection [7, 8]. In addition, Th17 cells and IL-17 have been reported to be involved in the pathogenesis of MTB [9]. IL-17 is a proinflammatory cytokine produced by Th17 cells and by airway structural cells, which provides IFN-*γ*-dependent or IFN-*γ*-independent protection to MTB infection (see Figs. 1 and 2) [10–12].

Indeed, in models of MTB infection, IL-17 and Th17 cells were first implicated in the protective immune response to rapidly growing extracellular bacteria in the lung and gut mucosal surfaces through efficient induction of neutrophil recruitment and tissue repair [13–15]. IL-17 and Th17 cells are important during the initial stages of infection and act upon hematopoietic and non-hematopoietic cells to promote the secretion of antimicrobial peptides such as G-CSF and CXC chemokines. As a consequence of this, DCs migrate to the local lymph nodes and induce the differentiation of both Th1 and Th17 cells. The increased levels of chemokines in the infected lung also promote recruitment of other protective cells such as macrophages and PMN and the formation of mononuclear granulomas. Moreover, an accumulation cytokines such as IL-6 and IL-23 in the lungs can further induce the differentiation and activation of Th17 cells and accelerate the pathogenesis of TB [16].

In this review we focus on the role of signal transduction pathways which have an impact on the pathogenesis of TB. Among these, the generation of ROS and the later activation of PPRs including TLRs and of the inflammasome are highlighted.

## Role of Reactive Oxygen Specious (ROS) in Pathogenesis of TB

Reactive oxygen species (ROS) and reactive nitrogen species (RNS) are considered to play important role in the pathogenesis of various inflammatory diseases [17, 18]. Under physiologic conditions; ROS are generated as byproducts of oxygen metabolism [17]. ROS are found in all biological systems and originate from the metabolism of molecular oxygen (O_2_). Under physiological conditions O_2_ undergoes reduction by accepting four electrons which results in the formation of water [18]. During this process, reactive intermediates such as the superoxide anion (∙O_2_), hydrogen peroxide (H_2_O_2_) and hydroxyl (∙OH)^−^ radicals are formed [19]. Activated macrophages express two major enzymes, phagocyte oxidase (NOX2/gp91^phox^) and inducible nitric oxide synthase (NOS2), which are able to generate reactive oxygen intermediates (ROI) and reactive nitrogen intermediates (RNI), respectively. Upon phagocytosis, the preformed NOX2 subunits assemble into an enzymatically active enzyme complex that transfers electrons across the membrane from cytosolic NADPH to molecular oxygen. This produces ∙O_2_ which dismutate into hydrogen peroxide (H_2_O_2_) and thus generate ∙OH radicals which are toxic to MTB [20]. Following inhalation of MTB, alveolar macrophages engulf the bacilli and initiate their killing using a number of mechanisms including the generation of ROI and RNI [21, 22].

The rapid generation of ROS is critical in host defense against many bacteria and fungi, and ROS has broad signaling functions [23]. For example, the NADPH oxidase protein complexes generate the superoxide anion and downstream ROS. NADPH oxidase is the principal source of ROS generation in activated neutrophils and macrophages. Thus, NADPH oxidase has an important role in host defense against MTB and any patients with a loss of function mutation in genes encoding components of this enzyme complex could be deficient in killing bacilli. Indeed, mutations in the CYBB gene encoding the gp91 (phox) subunit of the phagocyte NADPH oxidase is associated with MTB [21]. In addition, a hemizygous splice mutation in intron 5 of CYBB was linked to the concomitant occurrence of chronic granulomatous disease (CGD) with MTB [24].

IFN-γ induces NOS2 and its product nitric oxide (NO) which in turn can be broken down to nitrite and nitrate. Under acidic conditions, such as within the phagosomes of IFN-γ activated macrophages, nitrite forms nitrous acid, which dismutates to NO and the toxic radical, nitrogen dioxide [25]. NO can synergize with superoxide, produced by the macrophage or generated as byproduct of respiratory metabolism by the pathogen, to form the highly poisonous peroxynitrite (ONOO^−^) radical [26]. These ROI and RNI react with a wide range of molecules, including nucleic acids, proteins, lipids and carbohydrates, resulting in the killing of MTB. To counteract these actions, MTB uses a variety of molecules to either detoxify ROI and RNI before they can inflict damage or to repair the damage they cause [27]. In particular, the presence of MTB results in glucose-6-phosphate (G6P) being oxidised by NADP-dependent and F420-dependent (FGD1) dehydrogenases to generate NADPH, an important source of electrons, and thereby overcome oxidative stress [28]. In addition, MTB uses a combination of its cell surface alpha-ketoacid dehydrogenase complexes to form a NADH-dependent peroxidase and peroxynitrite reductase [29].

## Role of Toll Like Receptors (TLRs) in Pathogenesis of MTB

Pattern recognition receptors (PRRs) are a group of receptors which sense the presence of bacteria, fungi and viruses. PRRs are also responsible for recognizing endogenous molecules released from damaged cells, which are named damage associated molecular patterns (DAMPs) [30, 31]. To date, four different types of PRR families have been characterized [32]. These families include transmembrane proteins such as the TLRs and c-type lectin receptors (CTLRs), as well as cytoplasmic proteins such as the Retinoic acid-inducible gene (RIG)-I-like receptors (RLRs) and NOD-like receptors (NLRs) [33].

TLRs are a family of single membrane-spanning receptors of which 10 have been characterized in man and 13 in mouse [34–36]. TLRs play a critical role in both innate resistance and the initiation of adaptive immunity to infectious agents [37–40]. They act by recognizing pathogen-associated molecular patterns (PAMPs) or endogenous inflammation-associated molecules [36, 41, 42]. These are distinct molecular structures on microbes and different sets of TLRs have been associated with the response to different classes of microorganisms e.g. recognition of viruses by TLR3, TLR7, TLR8 and TLR9 [36, 41, 43–46].

Bacterial DNA which contains unmethylated CpG oligonucleotides (ODN) motifs also acts as important regulators of human neutrophil functions via TLR9. For example, stimulation of the TLR9 pathway by CpGODN induces CXCL8 production by human neutrophils via the generation of ONOO^−^ [47, 48]. TLR-ligand binding can induce two signaling pathways, the myeloid differentiation primary response gene 88 (MyD88)-dependent and MyD88-independent pathways, which induce the production of both pro-inflammatory cytokines and type I IFNs [36, 49, 50]. MyD88 is used by all TLRs except TLR3. These two distinct responses are mediated via the selective use of adaptor molecules recruited to the Toll/IL-1 receptor (TIR) domains of TLRs after ligand binding. Four adaptor molecules have been identified to date: MyD88, TIR-associated protein (TIRAP), TIR domain-containing adaptor protein-inducing IFN-β (TRIF) and TRIF related adaptor molecules (TRAM) [51].

MyD88 and TIRAP are responsible for the induction of pro-inflammatory genes, and TRIF and TRAM induce IFNs. In MyD88-dependent signaling, MyD88 is recruited to, and associates with, the cytoplasmic domain of the TLRs upon ligand binding. Then IL-1R-associated kinase 4 (IRAK-4) and IRAK- 1 are subsequently recruited and activated by phosphorylation. Activated IRAK-4 phosphorylates IRAK-1, which then, in turn, associates with tumor necrosis factor receptor (TNFR)-associated factor 6 (TRAF6). TRAF6 activates transforming growth factor (TGF) activating kinase 1 (TAK1) [36], which, in turn, phosphorylates IKK-β and mitogen-activated protein kinase (MAPK) kinase6 (MKK6), leading to degradation of I-κB, nuclear translocation of NF-κB and induction of inflammatory genes [52].

As a result TLR activation upregulates the transcription of proinflammatory cytokines including IL-1β, TNF-α and IL-6 which are essential for the recruitment of immune cells to the site of infection and controlling MTB infection [53–55]. Activation of the MyD88-dependent pathway also results in the activation of mitogen-activated protein (MAP) kinases (MAPK) such as p38 and JNK, which leads to the activation of AP-1 [56]. During MyD88-independent signaling TLR4 activation triggers the induction of a type 1 IFN response, leading to the induction of IFN-α and IFN-inducible genes [55]. The TLRs known to be involved in recognition of MTB are TLR2, TLR4, TLR9, and possibly TLR8 [36, 41, 43–46]. Four primary immunodeficiencies (PIDs) involving mutations in *MyD88*, *IRAK4*, *NEMO* and *IKBA* are associated with altered susceptibility to *M. tuberculosis* [57–59].

TLR2 can form heterodimers with both TLR1 and TLR6. These heterodimers have been implicated in the recognition of mycobacterial cell wall glycolipids including lipoarabinomannan (LAM), lipomannan (LM), 38-kDa and 19-kD mycobacterial glycoproteins, phosphatidylinositol mannoside (PIM), triacylated (TLR2/TLR1) or diacylated (TLR2/TLR6) lipoproteins [49, 60, 61]. TLR2 and TLR1 act together to mediate responses to *M. tuberculosis* [62, 63] and the role of TLR1/2 gene variants in the predisposition to tuberculosis has been investigated. Most studies have focused on TLR2 variants and only weak and non-replicated associations have been reported to date [62, 63]. TLR2 is believed to be important in the initiation of the innate host defense against MTB [61, 64]. In addition, IL-1*β* production is dependent upon TLR2 and TLR6, but not TLR4 or TLR9, stimulation [65]. TLR2 is also important for IL-12 release in macrophages, but not in DCs [66]. TLR2^−/−^ mice show defective granuloma formation following MTB infection and have a greatly enhanced susceptibility to infection compared to the WT mice [53, 67]. In addition, TLR2^−/−^ mice are unable to control chronic infection with MTB [67, 68]. Mice lacking TLR9 also succumb earlier to MTB infection than wild-type animals [61, 66, 69–72].

The role of other TLRs, such as TLR4 and TLR9 in the pathogenesis of MTB has not studied in such detail [65, 73, 74]. Mice deficient in the TLR/IL-1R family receptor adaptor molecule MyD88 have been shown to be highly susceptible to infection with MTB, which suggests a major role for this pathway in the innate defense against the MTB [68, 75–86]. In addition, TLR2-induced ROS production plays a crucial role in the expression of CXCL8 and CCL2 in human monocytes requiring the activation of both p38 and ERK1/2 MAPK pathways [78]. Overexpression of both TLR2 and TLR4 are important for viable MTB infection in human cell lines [79].

Other studies in mice with inactivated TLR genes indicated that TLR2 is important in controlling and surviving MTB infection [68, 73]. However, other studies suggested that TLR4 is critical for surviving MTB infection [80, 87]. The importance of TLR4 may depend on the dose of MTB used for challenge [88] or the mouse strain used [81]. Human studies show that polymorphisms in both TLR2 and TLR4 are associated with increased susceptibility to microbial infections possibly by changing the Th1/Th2 response [82–85]. Interestingly, Fenhalls et al. reported that the expression of TLRs in TB lung granulomas related to the presence or absence of immunohistologically detectable IL-4 [86].

Changes in TLR expression and/or their down-stream activation state might represent useful markers of the immunological status of TB patients and their contacts. The TLR distribution in TB granulomas lesions indicates that TLR1, TLR2, and TLR4 are expressed in both immune cells and non-immune cells; however TLR9 is only detectable in immune cells [89]. Furthermore, in an animal model of TB, TLR8-deficient mice succumb more rapidly to infection with *M. tuberculosis*, despite efficiently controlling the number of viable bacilli in different organs. Although no changes in CD4+ and CD8+ T-cells were observed there were increases in lung neutrophils and macrophages. Exaggerated mortality was due to massive liver necrosis and was reversed by a combination of blocking antibodies to IL-1 and TNF-α. Thus, in this model of MTB infection, TLR8 plays a key role in dampening inflammation and tissue damage [90]. Overall, Recognition of MTB by TLRs triggers various intracellular signaling cascades ultimately resulting in the production of cytokines, chemokines and antimicrobial molecules [91, 92].

In humans, the association of TLR polymorphisms with susceptibility to TB remains to be confirmed [92]. Different polymorphisms in the human TLR2 gene were reported to associate with increased susceptibility to TB in some studies [93–97] but not others [98–101]. Furthermore, a MAL/TIRAP functional variant, affecting signaling through TLR2, was shown to be protective in TB [102]. Genetic polymorphisms in TLR4 were linked to an increased susceptibility and severity of pulmonary TB in an Asian population in India [103] but not in Indian or Chinese TB patients in Gambia [101, 102, 104]. This discrepancy might be due to a dynamic host-pathogen interplay between genetic and pathogen phenotypes [102].

## Role of Pentraxin 3 (PTX3) in Pathogenesis of MTB

Pentraxin 3 (PTX3), or TNF-stimulated gene 14 (TSG-14), is a 42-kDa soluble pattern-recognition receptor produced by phagocytes and non-immune cells at sites of inflammation or injury and plays an important role in female fertility and vascular biology [103]. PTX3 shows up to 28 % sequence identity to human C-reactive protein (CRP) and serum amyloid P-component (SAP) [104, 105]. It is a member of the pentraxin family which are involved in the acute phase response to injury, trauma and infection [106]. PTX3 is rapidly secreted into the serum of mice and humans from extra-hepatic sources after LPS, IL-1 or TNF-α stimulation [107].

PTX3 binds to the complement component C1q [108] and to microorganisms, including *Pseudomonas aeruginosa*, *Salmonella typhimurium* and *Aspergillus fumigates to* induce innate immune responses [109, 110] and to drive a protective adaptive immunity [111]. Since whole mycobacteria and mycobacterial lipoarabinomannan strongly induce PTX3 production by human mononuclear phagocytes [112] a role for PTX3 in the immunobiology of mycobacterial infection has been inferred. Interestingly, PTX3 receptor gene variants are associated with an increased risk of pulmonary tuberculosis in West Africans [113]. Furthermore, PTX3, levels are significantly correlated with the severity of clinical presentation at diagnosis and of lung involvement in disease and may represent a good biomarker for inflammation and disease activity during MTB infection [112].

## Role of Inflammasome Signaling in MTB

There are two classes of innate immune receptors described: **a)** TLRs, located on cell membranes or intracellularly, and **b)** NLRs located in the cytoplasm [114–118]. Both classes of receptors are programmed to recognize microbial PAMPs and danger-associated molecular patterns (DAMPs) and switch cells for activation to releasing of proinflammatory and chemokines. The importance of two receptors in pathogenesis of chronic lung disease has elicited much attention [119, 120]. In the next section, we describe the regulation of inflammasome signaling and discuss whether abnormalities in NLRP3 inflammasome function may be associated with MTB.

The inflammasome consists of a multimeric cytosolic complex comprising the adaptor protein apoptosis associated speck-like protein containing a caspase recruiting domain (ASC), a sensor protein such as NRLP3 together with the effector proteins caspase-1 and caspase 5 [119]. Several NLRs function in immunity through the formation of a multi-protein complex known as an inflammasome which play critical roles in the 0 pathogenesis of chronic disorders [119–121].

NLRs exist in three families; the NODs, the NLRPs and the (IPAFs). Stimulation of cells with PAMPs, or by DAMPs, leads to increased expression of IL-1β and other IL-1 cytokine family members, such as IL-18 and IL-33 [120]. Proinflammatory cytokines of the IL-1 family may play an important role in anti-mycobacterial host defense mechanisms [121]. Moreover, MTB stimulates inflammatory cells to release IL-1β through pathways involving TLR2/TLR6 and NOD2 receptors [122]. Recognition of MTB by TLR and NOD2 leads to increased transcription of pro-IL-1β through mechanisms involving ERK, p38 and Rip2, but not JNK. Interestingly, although caspase-1 is necessary for the processing of pro-IL-1β, activation of caspase-1 is not dependent on the stimulation of cells by MTB [123]. In human THP-1 macrophages, MTB activation results in secretion of IL-1β in an ASC/NLRP3-dependent manner [124]. In addition, Mycobacterium marinum activates IL-1β production in an NLRP3- and caspase-1-dependent manner in vitro highlighting the potential importance of inflammasome signaling in the pathogenesis of MBT [125, 126].

Inflammasome-mediated IL-1β secretion is triggered by a combination of signal transduction pathways activated via TLRs and purinergic (P2X7) receptors. In turn, IL-1β induces the release of GM-CSF which leads to the activation and increased survival of monocytes/macrophages and enhanced oxidative burst in the lungs, thus maintaining and prolonging inflammatory reactions [127]. The purinergic P2X7 receptor is the key driver of ATP-mediated inflammasome maturation and release of IL-1β [122, 128, 129]. Pro-inflammatory cytokine regulation by the inflammasome may be critical to long-term survival of MTB infection since experiments in IL-1α/β, IL-1R and IL-18 knockout (KO) mice have shown that these cytokines play a role in limiting bacterial burden in the lung, in regulating the subsequent expression of other cytokines, in controlling NO production and in the formation of granulomas [122, 128, 129].

Caspase-1 independent IL-1β production may also be critical for host resistance to MTB and this occurs independently of TLR signaling in vivo. Furthermore, although IL-1β induction by MTB in vitro depends on TLR triggering and the inflammasome, both triggers are dispensable for IL-1β production in mice infected with the pathogen in vivo [130]. Thus, although recent data established that IL-1β plays a critical component in innate resistance to *MTB*, the pathways involved in the expression and regulation of IL-1β induction following MTB infection in vivo are complex and may involve mechanisms that do not fit the classical paradigms of TLR recognition and inflammasome-mediated caspase-1 processing seen with other infections or in the response to MTB observed in vitro [130].

MTB-induced IL-1β secretion in human and mouse macrophages in vitro and this process was dependent on ASC, caspase-1, and NLRP3, but not NLCR4 [130]. In vivo, murine ASC helps protect the host from death during chronic MTB infection whilst the effects of Casp-1 and NLP3 were negligible. The inability of ASC KO mice to form organized granulomas and the reduced presence of lung dendritic cells indicates a breakdown in host defense against MTB. Thus, ASC was identified as a critical protein involved in the host response to MTB infection in an inflammasome-independent manner [75]. Other cytokines activated by the inflammasome have also been reported to play a role in the pathogenesis of MTB. Thus, IL-12 and IL-18 produced by dendritic cells and macrophages induce NK-cell activity and skew the immune response towards an IFN-γ-dependent Th1 response, which is considered critical for protection against MTB [8].

Data in MyD88-deficient mice which are highly susceptible to MTB and succumb very rapidly to infection supports a role for MyD88 in regulating MTB infection [75, 131]. MyD88, however, plays a role in both inflammasome and TLR signaling and this raises the possibility that a lack of IL-1β or IL-18 is responsible for the heightened susceptibility of MyD88 KO mice to MTB infection [122]. Fremond et al. reported that IL-1R-signalling is important for protection against MTB whilst IL-18R-signalling is not [128]. In contrast, Schneider et al has reported a similar degree of susceptibility to MTB infection to that observed in MyD88 KO mice in IL-18 KO mice [129]. IL-18 KO mice succumbed much more readily to experimental MTB infection than WT or TLR-2/-4 double KO (TLR-2/-4 DKO) mice. In the absence of IL-18, immunity to MTB was hampered by decreased Th1 responses and PMN-dominated lung immunopathology concomitant with unrestrained growth of the tubercle bacilli. Thus, some controversy still remains as to the precise role of IL-18 in the protective immunity against MTB infection [129].

## Conclusion and Future Outlook

TB remains one of the leading causes of death from a single infectious agent worldwide. In order to generate better protective strategies we need to further define the pathological mechanisms underlying the immune response to MTB. Whilst inflammasome and TLR cross talk does not seem to be essential for the primary control of MTB infection, recent data suggests a critical role of these pathways in the persistence of MTB. Activation of these pathways results in the release of inflammatory mediators that recruit protective cells to the infected area. However, there is a down side to this effect. Excessive production of IL-23 and IL-17 causes pathology due to excessive recruitment and phenotypic changes in inflammatory cells. Hence, there is a fine balance between Th1 and inflammasome/TLRs responses that is central in defining the outcome of MTB infection. The role and mechanisms underpinning PTX3 and other PPRs in the immune response to MTB still requires further elucidation however.

In addition, it is critical to further define the mechanisms associated with the cross talk between TLRs and the inflammasome and to use this knowledge to generate rational protective strategies that promote a balanced acquired immune response with minimal collateral damage. Determination of key nodes within the pathways involved in the pathogenesis of MTB may provide new therapeutic targets to prevent the persistence of disease.

## Abbreviations

APC: Antigen-presenting cell
BAL: Bronchoalveolar lavage
cDC: Conventional dendritic cell
COPD: Chronic obstructive pulmonary disease
CXCL: CXC-chemokine ligand
CTL: Cytotoxic T lymphocytes
CMR: Cell-mediated reactions
CLR: C-type lectin receptors
DTH: Delayed-type hypersensitivity
DCs: Dendritic cells
IFN-γ: Interferon gamma
IL: Interleukin
IPAF: IL-1β converting enzyme (ICE) protease activating factor
KO: Knockout
LAM: Lipoarabinomannan
LM: Lipomannan
LPS: Lipopolysaccharide
MTB: Mycobacterium tuberculosis
MMPs: Matrix metalloproteinase
NLRP: NACHT, LRR and PYD domain-containing protein
PTX3: Pentraxin 3
PAMPs: Pathogen-associated molecular patterns
pDC: Plasmacytoid dendritic cell
PMN: Polymorphonuclear cells
PRRs: Pattern recognition receptors
ROS: Reactive oxygen species
TLR: Toll-like receptor
TB: Tuberculosis
